# Addressing Barriers to HIV Point-of-Care Testing in Community Pharmacies

**DOI:** 10.3390/pharmacy9020084

**Published:** 2021-04-16

**Authors:** Kimberly McKeirnan, Sorosh Kherghehpoush, Angie Gladchuk, Shannon Patterson

**Affiliations:** Pharmacotherapy Department, College of Pharmacy and Pharmaceutical Sciences, Washington State University, Spokane, WA 99202, USA; s.kherghehpoush@wsu.edu (S.K.); angelina.gladchuk@wsu.edu (A.G.); shannon.k.patterson@wsu.edu (S.P.)

**Keywords:** human immunodeficiency virus (HIV), point-of-care testing, community pharmacy practice

## Abstract

Significant numbers of human immunodeficiency virus (HIV) infections are transmitted unknowingly, making efforts to increase HIV testing accessibility crucial. As trusted healthcare providers, pharmacists can increase accessibility of HIV screening and referral services. However, challenges with lack of private counseling and testing space, need for training and education, lack of adequate staffing, heavy workload, and uncertainty supporting patients with reactive results have been previously reported by community pharmacists as barriers to offering HIV screening. The objective of this study was to investigate pharmacists’ opinions of strategies for addressing these barriers. A survey was developed to gather information regarding steps that could be taken to increase pharmacist comfort and interest offering HIV point-of-care testing (POCT) services. Thirty pharmacies were contacted and representatives from twenty-six responded. Pharmacists reported that they were likely or very likely to offer HIV POCT if they were given the following: a 2 h training session on administering and interpreting HIV POCT (73%); a 4 h education session on a variety of HIV education topics (73%); training about couples testing, post-test counseling, and de-escalation techniques (58%); or a semi-annual CE training (58%). Pharmacist respondents were likely or very likely (81%) to implement HIV POCT if there was a protocol in place so that patients with a reactive screening would out be referred for diagnostic testing and if there was a script provided as a template for post-test counseling (81%). The majority of pharmacists (69%) also preferred the appointment-based model rather than a walk-in or combination option and preferred (77%) having 20–30 min of dedicated time with the patient to provide adequate testing, education, and counseling. By using these strategies to improve comfort and likelihood implementing HIV POCT, pharmacists can increase access to HIV testing and decrease the spread of HIV.

## 1. Introduction

As of 2018, the Center for Disease Control and Prevention (CDC) reports that an estimated 1.2 million individuals in the U.S. were infected with human immunodeficiency virus (HIV) [1]. It is estimated that one in seven individuals with HIV are currently unaware of their HIV infection [1]. Although there is no cure for HIV, identification of infection and immediate treatment with antiretrovirals is recommended to suppress HIV viral load and preserve immune function, allowing HIV to be treated as a chronic disease state [2]. Symptoms associated with HIV can be non-specific, which highlights the importance for routine testing in order to reduce community transmission [3]. Currently, the CDC recommends that all individuals aged 13–64 receive routine HIV testing but estimates that nearly 45% of adults in the U.S. have never been tested [1].

Individuals at an elevated risk for acquiring HIV include those who are experiencing homelessness, members of the lesbian, gay, bisexual, transgender and queer or questioning (LGBTQ) community, racial and ethnic minorities, and those who struggle with substance abuse [1,4,5,6]. New cases of HIV in persons aged 13 years and older in 2018 were estimated to have been transmitted by male-to-male sexual contact (67%), heterosexual contact (23%), and injection drug use (7%) [1,6]. Moreover, the HIV incidence rate was highest in Blacks/African Americans and second highest in Hispanics/Latinos [1].

Novel strategies are being implemented to increase HIV testing and referral to care for all patients. One such strategy is HIV point-of-care testing (POCT) offered in retail clinics and community pharmacies [7]. Pharmacists are among the most trusted and accessible healthcare providers, with more than 60,000 pharmacy locations in the U.S and 13 billion annual visits [8,9]. The CDC estimates that 90% of urban populations live within two miles of a pharmacy and 70% of rural populations live within 15 miles of a pharmacy [10]. The accessibility and trust that pharmacists develop with their patients allows them to be uniquely positioned to have a significant impact on the spread of HIV.

The Clinical Laboratory Improvement Amendments of 1988 (CLIA) allow pharmacists to apply through Centers for Medicare and Medicaid (CMS) and be approved for a CLIA certificate of waiver [11,12]. Once the waiver is in place, pharmacists may implement POCT, including rapid HIV POCT, as long as state-specific regulations are met. HIV Point-of-Care tests detect antibodies with or without antigens to the HIV virus depending on the product used. The CDC recommends using a window period of 90 days for HIV antibody tests meaning that if in an individual was exposed within the last 90 days, the POCT result may not be reliable. Although these tests have a high degree of sensitivity and specificity, a reactive result is still considered a preliminary positive that needs to be confirmed with laboratory assays for diagnosis. Individuals with a reactive screening can be referred to local primary care providers or clinics for confirmatory testing and treatment initiation. Individuals with a non-reactive HIV POCT and risk factors for acquiring HIV as outlined by the CDC, may also be referred or prescribed pre-exposure prophylaxis (PrEP) by the pharmacist under a collaborative drug therapy agreement (CDTA). Pharmacy-led rapid HIV testing with same-day results has been shown to reduce follow-up and overall costs, and increased linkage to care [13,14,15].

As of March 2020, there were over 5700 facilities with CLIA certificates of waiver for POCT in Washington state and of those facilities, over 650 were pharmacies [16]. Although many state laws are currently in support of HIV POCT, the majority of CLIA-certified pharmacies do not yet offer these services [17]. Previous studies have identified a lack of infrastructure for private counseling and testing, additional need for HIV training and education, lack of adequate staffing, heavy workload, and uncertainty supporting patients with reactive results to be significant barriers for pharmacists to offer HIV testing in community pharmacies [18,19,20]. To increase patient access to screening, addressing these barriers is key. However, research going beyond identifying barriers and instead working toward addressing these barriers for community pharmacists has not been explored. According to the Consolidated Framework for Implementation Research, (CFIR) assessing both barriers and strategies or facilitators to overcome those barriers can provide a practical guide for implementing innovative services, such as HIV POCT in community pharmacies [21]. Thus, the objective of this study is to investigate pharmacists’ opinions of strategies for addressing previously reported barriers to implementation of HIV testing services in a community pharmacy setting.

## 2. Materials and Methods

Methods used in this study were determined to satisfy the criteria for Exempt Research by the Washington State University Human Research Protection Program (Institutional Review Board# 18225).

### 2.1. Survey Development

A survey was developed to gather information regarding steps that could be taken to increase pharmacist comfort and interest offering HIV POCT and services based on previously reported barriers. Five-point Likert-scale questions and free-response questions were included. Survey questions looked to address topics previously reported as barriers to implementing HIV POCT in community pharmacies and condensed into four domains: (1) pharmacist comfort; (2) pharmacist training; (3) pharmacy logistics and preparedness; and (4) pharmacy protocols and referral pathways [18,19,20]. After the initial survey was developed, it was piloted by two certified HIV pharmacists who also have experience in a community pharmacy setting. Suggestions from the HIV-certified pharmacists were incorporated. The final draft of the survey was limited to 21 questions because the research team wanted to ensure it would take less than 10 min to complete over the phone. The survey tool with questions organized by domain is displayed in Table S1.

### 2.2. Participants

Potential pharmacies to include in the phone survey were identified by performing a Provider Credential Search using the Washington State Department of Health website to identify all licensed pharmacies in Spokane County. The state of Washington was chosen because this state has a progressive pharmacy practice act allowing pharmacists to prescribe and dispense PrEP and PEP under a CDTA. Specifically, Spokane, Washington was chosen because recent interest in implementing HIV POCT testing was expressed at a recent community pharmacy association meeting. The list of pharmacies resulting from the search was then reviewed to remove pharmacies that were in an institutional setting, were no longer in business, or did not appear to dispense medication directly to patients. In instances where several pharmacies from within the same chain were identified, the student researchers selected two pharmacies in separate geographical areas of Spokane county to survey. These methods resulted in a list of 30 pharmacies identified to contact for participation in the survey.

### 2.3. Data Collection and Analysis

Surveys were conducted verbally via phone by two student pharmacist researchers (AG, SP) under the supervision of a pharmacist faculty member with experience conducting survey research (KM). Both student pharmacists had experience working as pharmacist interns but did not have a prior relationship with any of the study pharmacies they contacted. The researchers called each pharmacy during normal business hours and asked to speak to the pharmacist. The researchers explained that they were conducting a research project, the survey would take approximately 5–10 min, participation in the survey was voluntary, and individually identifying information would not be collected. Some pharmacists indicated that they were not available at the time of initial contact and had suggested a call-back time that better fit their schedule, in which case a follow-up phone call was made at their convenience. The researchers documented the pharmacists’ responses in Microsoft Excel. The pharmacy name was documented to ensure that all of the pharmacies identified were contacted, but the name of the individual pharmacist responding was not recorded. Once the surveys were completed, the data was aggregated and analyzed in Microsoft Excel using descriptive statistics.

## 3. Results

Thirty pharmacies were contacted to complete the 21-item phone survey. Pharmacists from 26 of the 30 pharmacies agreed to participate. Four pharmacists (all from chain pharmacies) declined to participate and reported lack of time as the primary issue, even after attempts to call back at a more convenient time. Of the 26 participating pharmacies, four were independent pharmacies and 22 were chain pharmacies.

**Figure 1:**
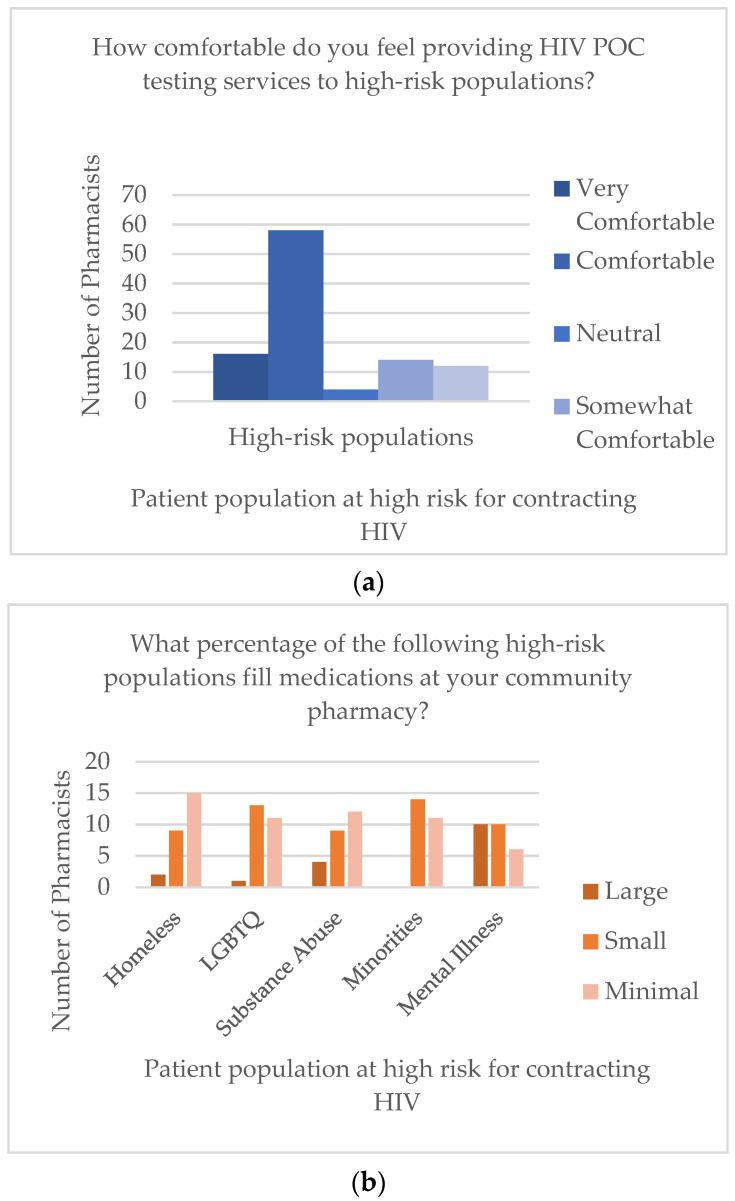
Responses to Question 2 (**a**) and Question 3 (**b**) regarding populations at high-risk for contracting HIV [1,4,5,6].

Of the 26 participating pharmacies, three were self-reported as high volume (defined as 400 or more prescriptions filled per day), 13 as medium volume (200–400 prescriptions filled per day), and 10 as low volume (less than 200 prescriptions filled per day). Results from questions regarding percentages of patients in high-risk populations (Question 2) and comfort providing HIV POCT to these populations (Question 3) are displayed in Figure 1. Responses to Likert-scale questions are displayed in Table 1.

Question 9 asked participants what additional training would help them feel comfortable providing HIV POCT. The most common responses included substantially more training about HIV in general, hands-on experience with testing, and support from corporate leadership. Full results are shown in Table 2.

Question 14 asked participants to quantify the time needed to conduct the test, discuss results, and provide education and counseling for a single patient if the test itself would take 5 min. The most popular response was 20 to 30 min (Table 2). Participants were also asked their preferred method of providing HIV POCT among the options of appointment-based testing only, walk-in, or both (Question 15). The majority (*n* = 18, 69%) preferred appointment-based testing only, followed by both appointment-based and walk-in options (*n* = 7, 27%), and walk-in only (*n* = 1, 4%).

Question 16 asked participants to identify additional challenges to implementing HIV POCT in their pharmacy beyond the issues of staffing time and physical space. Pharmacists reported corporate support for implementation, adding the testing into workflow, and other issues as challenges for implementing HIV POCT in their own pharmacies, as shown in Table 2.

Finally, participating pharmacists were asked if they had any concerns regarding referring patients with a reactive screening test and collaborating with community partners (Question 21). The most common concern reported was ensuring patient follow-up and minimizing the number of falling out of care (Table 2).

## 4. Discussion

This study sought to identify solutions to specific factors historically reported by pharmacists as barriers in providing HIV POCT. The goal of this work was to consider strategies to address or minimize these barriers by increasing comfort providing HIV POCT and the likelihood of implementing these services in the community pharmacy setting.

The first two domains identified from previous studies as barriers to implementation of HIV POCT in pharmacies were pharmacist level of comfort and training. Although POCT services are increasingly being incorporated into the doctor pharmacy curriculum, many pharmacists believe they are inadequately trained to confidently provide these services [20] and have a low level of comfort providing POCT [19,20,22]. To address this, pharmacists interviewed in this study were asked to rate their comfort level offering HIV POCT in their community pharmacy if they participated in different types of training on this topic. Pharmacists reported that they were likely or very likely to offer HIV POCT if they were given the following: a 2 h training session on administering and interpreting HIV POCT (73%); a 4 h education session on a variety of HIV education topics (73%); training about couples testing, post-test counseling, and de-escalation techniques (58%); or a semi-annual CE training (58%). When asked what training would help them feel comfortable providing HIV POCT, 7 out of 26 (27%) commented they wanted more training about HIV in general. By offering new programing or taking advantage of existing educational opportunities about HIV-related topics and POCT administration, the issue of pharmacist comfort offering POCT could be readily addressed. Another concern raised by pharmacists about comfort offering HIV POCT is having a conversation with patients about a reactive screening and ensuring they have access to diagnostic testing. Pharmacists in this study reported that they were likely or very likely (81%) to implement HIV POCT if there was a protocol in place so that patients with a reactive screening would be referred for diagnostic testing and if there was a script provided as a template for post-test counseling (81%).

Another relatively new service in pharmacy is the identification of high-risk patients and the ability of pharmacists to furnish pre-exposure (PrEP) and post-exposure (PEP) prophylaxis by pharmacists in California (Senate Bill No. 159) [23]. Pharmacists in this study reported that they were likely or very likely to implement prescribing and dispensing of PrEP (81%) if they were offered training and education about identifying high-risk patients. Populations at higher risk for acquiring HIV include those experiencing homelessness, struggling with substance abuse disorder, members of the LGBTQ community and racial/ethnic minorities [1,4,5,6]. Survey respondents were generally comfortable or very comfortable (70%) providing HIV POCT to high-risk populations and indicated that a standardized protocol for the identification of PrEP eligibility would increase the likelihood of implementing PrEP prescribing and dispensing services in their pharmacy. Training pharmacists to identify eligible patients and offer PrEP could increase access and decrease the spread of HIV among those who are at highest risk.

Issues related to staffing and the time commitment required to perform clinical services were at the forefront of respondents’ concerns. When asked about the time requirement to provide a single patient an HIV screening, the majority of respondents (77%) chose 20 to 30 min or more to provide adequate testing, education, and counseling. In a community pharmacy setting it can be quite difficult to identify a 20–30 min window where a pharmacist engaged in workflow could focus solely on one task with one patient. Although there are rapid HIV POCTs that give results within a 5 min time frame, effective education, counseling, and discussion of next steps for a patient can be time consuming and could vary from patient to patient depending on engagement and screening results. Not surprisingly, over 80% of surveyed pharmacists were likely or very likely to implement HIV POCT services if there was a pharmacist position dedicated to solely providing clinical services such as POCT.

The majority of pharmacists (69%) also preferred the appointment-based model rather than a walk-in or combination option. Although the appointment-based model eases the burden on pharmacy workflow and allows for a more structured patient-care model, one of the most attractive aspects of pharmacist-provided clinical services in the community setting can be the immediate accessibility and availability of a healthcare provider to the patient. If an appointment is needed for the HIV POCT, is this truly more accessible than an appointment in a physician’s office? In order to balance pharmacist availability and patient convenience, the barrier of time to offer these services needs to be addressed. In a 2017 study by Young and Bendavid evaluating the relationship between HIV testing, stigma and health service usage, researchers discovered that patients often attempt to conceal their desire for HIV testing and are less likely to list HIV testing as a primary reason for a visit with a healthcare professional [24]. To minimize stigma, promote early diagnosis and treatment and increase patient uptake of HIV testing services, pharmacies may consider implementing HIV POCT using the CDC’s recommended “Opt-out” approach where routine HIV testing is standard practice unless a patient specifically declines [25]. This would potentially lead to a streamlined HIV testing service that can be economically beneficial and reduce the spontaneity of requests for testing services.

Another possible solution to the lack of staffing and availability of pharmacists to implement POCT in community pharmacy is to consider the expanding role of pharmacy technicians. Pharmacy technicians are taking on many advanced roles under the supervision of licensed pharmacists [21]. When asked about the likelihood of implementing HIV POCT services if pharmacy technicians were involved in the process, most responding pharmacists (65%) were likely or very likely to implement HIV POCT. A recent study by Hill et al. evaluated the number of health screenings offered in pharmacies where pharmacy technicians were involved in the POCT process compared with control pharmacies where technicians did not participate in this process [26]. The results showed that pharmacies with technician involvement in POCT offered 16.8% more screenings and also reported patient satisfaction with the screenings provided by the technicians [26]. For pharmacists who are considering implementing HIV POCT, adding technicians into the process may alleviate issues with pharmacist workflow and increase the number of screenings offered without compromising patient satisfaction.

In addition to the personnel-related concerns, community pharmacies often lack the physical space needed to perform clinical services. Many pharmacies provide a semi-private area to administer immunizations, but these may be insufficient when privacy is needed for 20–30 min or more for a private discussion of HIV screening results and effective education and counseling. Nearly 90% of surveyed pharmacists responded as likely or very likely to implement HIV POCT services in their community pharmacy if they had a private counseling room. In the setting of a reactive result, a private area dedicated to post-test counseling is a necessity. Although a reactive HIV POCT does not equate to a diagnosis of HIV, a preliminary positive result is unlikely to be welcomed and potential reactions may range from devastation, rage, or overwhelming guilt [27,28]. Creating private counseling rooms for HIV screening and consultation could increase the likelihood of successful implementation of HIV POCT and provide patients with a safe space that allows for comforting and an opportunity to ask questions.

There are limitations to this research. First, this survey had a response rate of 86% and included pharmacists from independent and chain pharmacies. However, data was collected in one city in a single state where many pharmacists are used to offering POCT for other conditions and prescribing under a CDTA. With these limitations in mind, the responses in this survey may not adequately reflect the opinions of pharmacists nation-wide, particularly in states where POCT and/or prescribing under a CDTA are uncommon. Additionally, the participants of this study were pharmacists who were employed by the identified pharmacy. We did not gather data on the specific role of the pharmacist (staff pharmacist, pharmacist in charge, float pharmacist) which could have led to a higher level of comfort with implementation. Our relatively small sample size and limited geographic variability may limit the generalizability of results. The results of this work will be utilized to develop additional qualitative research as the next step in this project. Key informant interviews and focus groups utilizing the principles of the CFIR [21] will allow the researchers to conduct a more in-depth exploration into the strategies identified to overcome previously reported barriers to HIV POCT.

Another limitation comes from Question 2, which asked participants to estimate the percentage of their patients from the populations previously reported to be high-risk for HIV. The intent of this question was to quantify the frequency with which these pharmacists would encounter patients at high risk for contracting HIV. If study pharmacists regularly engaged with patients at high risk for HIV, offering HIV POCT would arguably be even more important. However, in retrospect, the researchers believe it was difficult and also likely inaccurate for pharmacists to estimate how many patients they had in each of these groups based their perceptions alone. This question will not be included in future iterations of this work.

Finally, the results of the survey indicated a high degree of comfort in providing HIV POCT, which is in contrast to a previously published study [19], so repeating this survey with different study populations in separate communities would provide additional insight into this topic.

## 5. Conclusions

This study took previously reported barriers for implementing HIV POCT in a community pharmacy a step further by gathering pharmacists’ opinions about potential strategies for addressing these barriers. Results show that pharmacists are comfortable engaging with patients in high-risk groups and are willing to implement HIV POCT if issues of time and staffing, physical space in the pharmacy and pharmacist education and training are addressed. Strategies that participating pharmacists reported may increase their level of comfort and likelihood of implementation of HIV POCT included offering pharmacists training and education about HIV POCT administration, interpretation of results, post-test counseling, and de-escalation techniques; appointment of a pharmacist solely dedicated to providing clinical services such as POCT; engaging trained pharmacy technicians in the POCT process; implementation of an appointment-based model; allowing for 20–30 min per patient for HIV POCT; creation of a private counseling room; and a referral pathway for patients with a reactive screening. Offering training for pharmacists to identify patients who are eligible for PrEP and PEP dispensing could also increase the likelihood of pharmacists offering these services. Pharmacists, as accessible and trusted healthcare providers, are well positioned to increase access to HIV testing and decrease the spread of HIV by addressing these issues and offering POCT in community pharmacies.

## Figures and Tables

**Table 1:** Results from Likert-scale survey questions (numbers 4–8, 10–13, and 17–20).

Question	Very Likely *n* (%)	Likely *n* (%)	Neutral *n* (%)	Somewhat Likely *n* (%)	Not Likely *n* (%)	Omit *n* (%)
4. If you were offered a 2 h training session on how to administer the point-of-care testing (POCT) and accurately interpret the results, how likely are you to implement human immunodeficiency virus (HIV) POCT in your community pharmacy?	6 (23)	13 (50)	2 (8)	2 (8)	3 (11)	0 (0)
5. If you were offered a 4 h education session on HIV prevention and screening covering topics such as: Disease state overview, risk factors for transmission, special populations, and pre-exposure prophylaxis (PrEP), how likely are you to implement HIV POCT in your community pharmacy?	7 (26)	12 (46)	3 (12)	2 (8)	2 (8)	0 (0)
6. If you were offered training on couples testing, post-test counseling and de-escalation techniques, how likely are you to implement HIV POCT in your community pharmacy?	5 (19)	10 (39)	2 (8)	4 (15)	4 (15)	1 (4)
7. If you were offered semi-annual (every 6 months) continuing education (CE) training for HIV POCT, how likely are you to implement HIV POCT in your community pharmacy?	4 (16)	11 (42)	5 (19)	4 (16)	2 (7)	0 (0)
8. Pharmacists are increasingly gaining authority to prescribe Pre-Exposure Prophylaxis (PrEP) for individuals who are at high risk for acquiring HIV through Collaborate Drug Therapy Agreements (CDTA). If you were offered training and education in identifying high-risk patients who may benefit from the use of PrEP, how likely are you to implement prescribing and dispensing of PrEP at your pharmacy?	8 (31)	13 (50)	2 (8)	1 (4)	2 (7)	0 (0)
10. Pharmacists have historically reported a lack of staffing, pharmacist availability and physical space to provide clinical services in the community setting. If there was a pharmacist position solely dedicated to providing clinical services in your pharmacy (such as POCT, immunizations, etc.) how likely are you to implement HIV POCT in your community pharmacy?	13 (50)	8 (31)	3 (12)	0 (0)	2 (7)	0 (0)
11. If Pharmacy Technicians, with the appropriate training and education, were providing the entirety of the HIV POCT service, how likely are you to implement these services in your pharmacy?	3 (12)	11 (42)	3 (12)	0 (0)	9 (34)	0 (0)
12. If Pharmacy Technicians, with the appropriate training and education, were to administer the HIV POCT and refer to the pharmacist for interpretation, post-test counseling and referral, how likely are you to implement these services in your community pharmacy?	9 (34)	8 (31)	2 (8)	1 (4)	6 (23)	0 (0)
13. HIV carries many stigma-related concerns and thus a private area to provide HIV POCT services is necessary. If your pharmacy had a private counseling room, how likely are you to implement HIV POCT in your community pharmacy?	10 (38)	13 (50)	2 (8)	0 (0)	1 (4)	0 (0)
17. If there was a protocol in place for referral of patients who have a reactive HIV screening with specific local partners that offer confirmatory diagnostic testing, how likely are you to implement HIV POCT services in your community pharmacy?	10 (38)	11 (42)	3 (12)	0 (0)	1 (4)	1 (4)
18. If there was a protocol in place for referral of patients who require comprehensive post-test counseling or referral for PrEP, how likely are you to implement HIV POCT services in your community pharmacy?	8 (31)	13 (50)	4 (15)	0 (0)	1 (4)	0 (0)
19. If there was a standardized risk-determination questionnaire that would indicate whether the patient qualifies for PrEP, how likely are you to implement the prescribing/dispensing PrEP to high-risk patients?	12 (46)	8 (31)	4 (15)	1 (4)	1 (4)	0 (0)
20. If there was a standardized script provided for post-test counseling on reactive and non-reactive HIV POCT, how likely are you to implement HIV POCT services in your community pharmacy?	9 (35)	12 (46)	3 (11)	0 (0)	2 (8)	0 (0)

**Table 2:** Pharmacist responses to Questions 9, 14, 16, and 21.

Pharmacists’ Suggestions of Training that Would Help them Feel Comfortable Providing Human Immunodeficiency Virus (HIV) Point-of-Care Testing (POCT) (Question 9)
• Substantially more additional training about HIV in general (*n* = 7, 27%)
• Hands-on experience with testing (*n* = 5, 19%)
• Support from corporate leadership (*n* = 5, 19%)
• More staff/labor hours (*n* = 3, 12%)
• Assistance with development of a collaborative drug therapy agreement (CDTA) (*n* = 2, 8%)
• A detailed implementation plan (*n* = 2, 8%)
• Training on talking to couples about reactive results (*n* = 1, 4%)
• Training for de-escalation during emotional situations (*n* = 1, 4%)
• And a motivational interviewing refresher course (*n* = 1, 4%)
Pharmacists’ opinions of the amount of time needed to conduct the HIV POCT, discuss results, and provide education and counseling if the test itself takes 5 min (Question 14)
• 5 to 10 min (*n* = 1, 4%)
• 15 to 20 min (*n* = 4, 15%)
• 20 to 30 min (*n* = 14, 54%)
• 30 to 45 min (*n* = 4, 15%)
• More than 45 min (*n* = 2, 8%)
• Prefer not to answer (*n* = 1, 4%)
Challenges to implementing HIV POCT beyond staffing time and physical space identified by pharmacist participants (Question 16)
• Corporate support for implementation (*n* = 4, 15%)
• Adding HIV POCT into workflow (*n* = 3, 12%)
• Advertising the service to the community (*n* = 2, 8%)
• Cost of testing and potential for reimbursement (*n* = 1, 4%)
Concerns regarding referring patients with a reactive screening and collaborating with community partners identified by pharmacist participants (Question 21)
• Ensuring patients followed up with another provider and not “falling between the cracks” (*n* = 3, 12%)
• Cost of testing and insurance coverage for testing and pre-exposure prophylaxis (PrEP)/post-exposure prophylaxis (PEP) (*n* = 2, 8%)
• Obtaining liver and kidney function blood tests prior to beginning PreP/PEP (*n* = 1, 4%)
• Limitations of the pharmacy’s computer system to document encounters (*n* = 1, 4%)

## Data Availability

The data presented in this study are available on request from the corresponding author.

## Supplementary Materials

The following are available online at https://www.mdpi.com/article/10.3390/pharmacy9020084/s1, Table S1. Survey questions organized by domain.
