# Supplementary material for: Addressing Barriers to HIV Point-of-Care Testing in Community Pharmacies
Source: Pharmacy (Basel). 2021 Apr 16;9(2):84. doi: 10.3390/pharmacy9020084 (PMC8167761; doi:10.3390/pharmacy9020084)
Supplement: Supplementary file 1 [file pharmacy-09-00084-s001.zip › pharmacy-1156843-supplementary.zip!/pharmacy-1156843-SI.pdf]

pharmacy MDPI

Table S1. Survey questions organized by domain.

Question	Domain
1. How would you classify your pharmacy with respect to volume?	Pharmacy demographics
Pharmacists have historically reported a lack of comfort providing HIV POCT in a pharmacy setting. Questions 2 and 3 pertain to pharmacist's comfort providing care to populations at a higher risk for acquiring HIV.	
2. There are certain populations that are at higher risk for acquiring HIV than the general population. What percentage of the following high-risk populations fill medications at your community pharmacy?[1,4-6]	Pharmacist comfort providing HIV POCT
3. How comfortable do you feel providing HIV POCT services to these populations?	
Pharmacists have previously reported that adequate HIV training and education is necessary to successfully implement point-of-care HIV testing in the community setting. The questions 4 to 7 pertain to HIV training and education.	
4. If you were offered a 2-hour training session on how to administer the POCT and accurately interpret the results, how likely are you to implement HIV POCT in your community pharmacy?	
5. If you were offered a 4-hour education session on HIV prevention and screening covering topics such as: Disease state overview, risk factors for transmission, special populations, and pre-exposure prophylaxis (PrEP), how likely are you to implement HIV POCT in your community pharmacy?	
6. If you were offered training on couples testing, post-test counseling and de-escalation techniques, how likely are you to implement HIV POCT in your community pharmacy?	Pharmacist training for HIV POCT
7. If you were offered semi-annual (every 6 months) continuing education (CE) training for HIV POCT, how likely are you to implement HIV POCT in your community pharmacy?	
8. Pharmacists are increasingly gaining authority to prescribe Pre-Exposure Prophylaxis (PrEP) for individuals who are at high risk for acquiring HIV through Collaborate Drug Therapy Agreements (CDTA). If you were offered training and education in identifying high-risk patients who may benefit from the use of PrEP, how likely are you to implement prescribing and dispensing of PrEP at your pharmacy?	
9. What additional training would you require in order to feel comfortable or very comfortable in providing HIV POCT in your community pharmacy?	
Pharmacists have historically reported a lack of staffing, pharmacist availability and physical space to provide clinical services in the community setting. Questions 10 to 16 pertain to pharmacy logistics.	
10. Pharmacists have historically reported a lack of staffing, pharmacist availability and physical space to provide clinical services in the community setting. If there was a pharmacist position solely dedicated to providing clinical services in your pharmacy (such as POCT, immunizations, etc) how likely are you to implement HIV POCT in your community pharmacy?	Pharmacy logistics
11. If Pharmacy Technicians, with the appropriate training and education, were providing the entirety of the HIV POCT service, how likely are you to implement these services in your pharmacy?	
12. If Pharmacy Technicians, with the appropriate training and education, were to administer the HIV POCT and refer to the pharmacist for interpretation, post-test counseling and referral, how likely are you to implement these services in your community pharmacy?	
13. HIV carries many stigma-related concerns and thus a private area to provide HIV POCT services is necessary. If your pharmacy had a private counseling room, how likely are you to implement HIV POCT in your community pharmacy?	
14. HIV POCT can be time consuming as patients may have many urgent questions about their results, the disease state, risk factors, etc. How much time do you think is appropriate to provide adequate testing, education and counseling for a single patient if test results are available within 5 minutes?	
15. If you were to offer HIV POCT in your community pharmacy, which of the following would you prefer with regard to scheduling?	
16. In addition to what was mentioned above with regard to staffing and physical space, what additional challenges do you face in implementing HIV POCT services in your community pharmacy?	
Pharmacists have expressed hesitancy for providing HIV POCT services due to a lack of collaboration with community partners for referral and follow-up diagnostic testing. Questions 17 to 21 pertain to pharmacy protocols and referral.	
17. If there was a protocol in place for referral of patients who have a reactive HIV screening with specific local partners that offer confirmatory diagnostic testing, how likely are you to implement HIV POCT services in your community pharmacy?	Pharmacy protocols and referral pathways
18. If there was a protocol in place for referral of patients who require comprehensive post-test counseling or referral for PrEP, how likely are you to implement HIV POCT services in your community pharmacy?	

19. If there was a standardized risk-determination questionnaire that would indicate whether the patient qualifies for PrEP, how likely are you to implement the prescribing/dispensing PrEP to high-risk patients?
20. If there was a standardized script provided for post-test counseling on reactive and non-reactive HIV POCT, how likely are you to implement HIV POCT in your community pharmacy?
21. What additional concerns do you have with regard to referral and collaboration with community partners in providing HIV POCT services in your community pharmacy?

Abbreviations used: HIV: human immunodeficiency virus; POCT: point-of-care testing; CE: continuing education; PrEP: pre-exposure prophylaxis.
