# Supplementary material for: Addressing Barriers to HIV Point-of-Care Testing in Community Pharmacies
Source: Pharmacy (Basel). 2021 Apr 16;9(2):84. doi: 10.3390/pharmacy9020084 (PMC8167761; doi:10.3390/pharmacy9020084)
Supplement: Supplementary file 1 [file pharmacy-09-00084-s001.zip › pharmacy-1156843-tex.zip!/Definitions/logo-orcid-eps-converted-to.pdf]

iD
