# Supplementary material for: Addressing Barriers to HIV Point-of-Care Testing in Community Pharmacies
Source: Pharmacy (Basel). 2021 Apr 16;9(2):84. doi: 10.3390/pharmacy9020084 (PMC8167761; doi:10.3390/pharmacy9020084)
Supplement: Supplementary file 1 [file pharmacy-09-00084-s001.zip › pharmacy-1156843-tex.zip!/Definitions/pharmacy-1156843-g001.pdf]

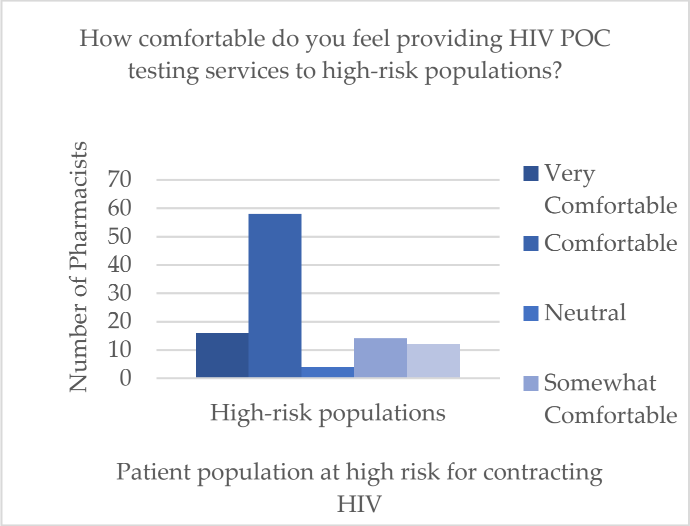
How comfortable do you feel providing HIV POC testing services to high-risk populations?
Number of Pharmacists
70
60
50
40
30
20
10
0
High-risk populations
Very Comfortable
Comfortable
Neutral
Somewhat Comfortable
Patient population at high risk for contracting HIV

(**a**)

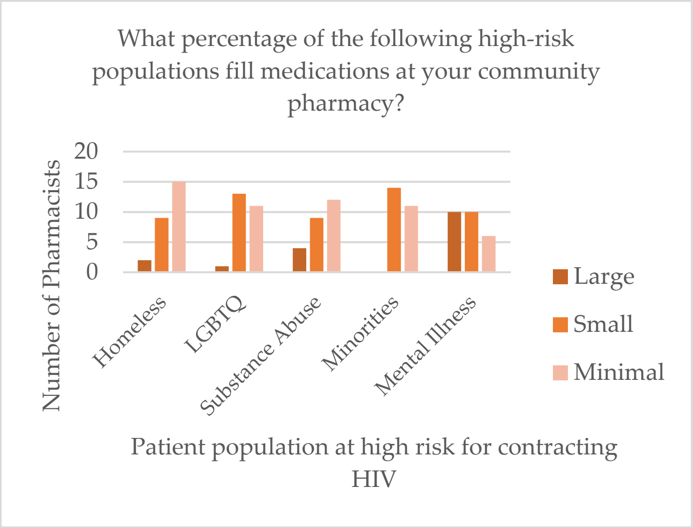
What percentage of the following high-risk populations fill medications at your community pharmacy?
Number of Pharmacists
20
15
10
5
0
Homeless
LGBTQ
Substance Abuse
Minorities
Mental Illness
Large
Small
Minimal
Patient population at high risk for contracting HIV

(**b**)
